# Genome-Wide Identification, Evolutionary, and Expression Analyses of Histone H3 Variants in Plants

**DOI:** 10.1155/2015/341598

**Published:** 2015-02-26

**Authors:** Jinteng Cui, Zhanlu Zhang, Yang Shao, Kezhong Zhang, Pingsheng Leng, Zhe Liang

**Affiliations:** ^1^College of Landscape, Beijing University of Agricultural, Beijing 102206, China; ^2^Shum Yip Group Limited, Shenzhen 518040, China; ^3^Department of Biological Sciences, National University of Singapore, Singapore 117543

## Abstract

Histone variants alter the nucleosome structure and play important roles in chromosome segregation, transcription, DNA repair, and sperm compaction. Histone H3 is encoded by many genes in most eukaryotic species and is the histone that contains the largest variety of posttranslational modifications. Compared with the metazoan H3 variants, little is known about the complex evolutionary history of H3 variants proteins in plants. Here, we study the identification, evolutionary, and expression analyses of histone H3 variants from genomes in major branches in the plant tree of life. Firstly we identified all the histone three related (HTR) genes from the examined genomes, then we classified the four groups variants: centromeric H3, H3.1, H3.3 and H3-like, by phylogenetic analysis, intron information, and alignment. We further demonstrated that the H3 variants have evolved under strong purifying selection, indicating the conservation of HTR proteins. Expression analysis revealed that the HTR has a wide expression profile in maize and rice development and plays important roles in development.

## 1. Introduction

Histones wrap DNA to form nucleosome particles that compact eukaryotic genomes [1]. Histone variants have evolved crucial roles in chromosome segregation, transcriptional regulation, DNA repair, sperm packaging, and other processes [2]. Histone H3 is one of the five main histone proteins involved in the structure of chromatin in eukaryotes. Histone H3 variants genes can be divided into two major groups: replication dependent and replication independent [3]. The replication dependent variants are highly expressed just before S-phase and then repressed at the completion of DNA replication. The replication-independent histone variants are constitutively expressed throughout the cell cycle [4]. In human the replication dependent variants are represented by H3.1 and the replication-independent variants best characterized are H3.3 and centromeric H3 variants (CenH3) [4]. H3.1 is similar in length and amino acid sequence to H3.3 except at few positions. CenH3 variants differ from the other H3 variants by a long extension of the N-terminal tail, which are not conserved among eukaryotes [5, 6]. Histone chaperones are escort factors associated with soluble histones involved in their transfer without being part of the final product [4, 7].

In animals, four amino acid substitutions distinguish H3.1 featuring A_31_-S_87_-V_89_-M_90_ from H3.3 featuring S_31_-A_87_-I_89_-G_90_ [8]. In plants, H3.1 and H3.3 are distinguished at positions 31, 87, and 90 and involve a different set of amino acids, implying that H3 variants evolved independently in plants and animals [5, 9], In addition, position 41 is a plant-specific substitution that discriminates H3.3 from H3.1 variants [6, 9]. Phylogenetic analyses also showed that H3.1 and H3.3 variants evolved independently, suggesting that H3 variants in plants and animals are analogous and result from convergent evolution [10].

There are 15 HTR genes coding for H3.1, H3.3, and CenH3 proteins in* Arabidopsis thaliana *[9]; H3.1 and H3.3 proteins play strikingly different roles. H3.3 enrichment positions are positively correlated with gene expression and to be biased towards the transcription termination site [11], which are common feature found in yeast,* Drosophila* and mammals [12–14]. The modification marks associated with transcriptional activity such as H3K27me and H3K36me are enriched in H3.3 in comparison to H3.1 [11].

Until now, genome-wide analyses of H3 variants have been conducted in* Arabidopsis* and several other plant species [6, 9, 11, 15]. However, comprehensive analyses of H3 variants in major plants are still lacking. Here, we studied the identification, evolutionary, and expression analyses of histone H3 variants. We searched for H3 variants in genomes representing a broad taxonomic sampling from distantly related plant evolutionary lineages, including eudicots, monocots, gymnosperm, lycophyte, bryophyte, and chlorophyte species. Subsequently, we classified the HTRs by phylogenetic analysis and alignment. We found the HTR genes in* Capsella rubella* might be a new class of H3 variants because of the unique sequence feature. Moreover, the selection and expression analysis suggested the functional conservation of H3 variants.

## 2. Methods

### 2.1. Sequence Retrieval

We performed BLASTP and TBLASTN searches among sequenced genomes of planta in Phytozome (http://www.Phytozome.net/) using* Arabidopsis* H3.1, H3.3, and CenH3 proteins as queries to identify HTRs (*e*-value < *e* ^−10^). The species represented a broad range of the plant lineages from unicellular green algae to multicellular plants (http://www.jgi.doe.gov/). We used a relatively strict criterion to collect HTRs with high-quality sequences. To verify the reliability of our results, each sequence was then searched against the protein conserved domain database (CDD) [16], SMART database [17], and the PFAM database [18], respectively. The detailed information (i.e., accession number, intron information) of all HTR sequences presented here is listed in Supplementary Table (see Supplementary Material available online at http://dx.doi.org/10.1155/2015/341598).

### 2.2. Construction of the Alignment

The HTR sequences were then aligned by MAFFT version 7 using the G-INS-i algorithm [19], followed by manual editing in MEGA 6.0 [20]. Only positions that unambiguously aligned were included in the further analyses. ProtTest 3.0 was used for amino acid substitution model selection using the Akaike Information Criterion (AIC) to choose the best-fitting tested model (LG + G) for phylogenetic analyses [21].

### 2.3. Phylogenetic Analyses

The neighbor joining (NJ) phylogeny was performed by MEGA version 6.0 with 1000 replicas; since LG + G is not available in MEGA, we use next available best model JJT + G model, and pairwise deletion. The maximum likelihood (ML) phylogeny was performed by PhyML software [22], with 100 replicas and the LG + G model.

### 2.4. Selection Analyses

Sequences used for selection analysis are listed in Supplementary Table. H3.1, H3.3, and CenH3 codon alignment was performed using MAFFT version 7 with the G-INS-i algorithm and then loaded into Hyphy [23] (along with a corresponding NJ phylogenetic tree). The HyPhy batch file NucModelCompare.bf with model rejection level of 0.0002 was used to establish the best fit of 203 general time-reversible (GTR) models of nucleotide substitution. The Hyphy batch file QuickSelectionDetection.bf was used to estimate site-by-site variation in rates.

### 2.5. Gene Expression Analysis

Maize and rice microarray-based datasets, with accession numbers GSE27004 [24] and GSE19024 [25], were downloaded from the NCBI Gene Expression Omnibus (GEO) [26]. A hierarchical cluster was created using the Cluster 3.0 [27] and viewed using the Java TreeView [28].

## 3. Result and Discussion

### 3.1. Identification of H3 Variants in Planta

**Figure 1:**
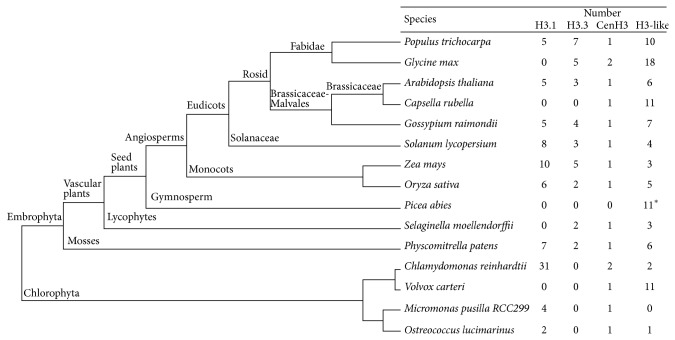
Phylogenetic relationships between all species investigated in this study. The total number of histone three related (HTR) genes found in each genome is indicated on the right. Data was obtained from the phytozome (http://www.phytozome.net/) and Norway spruce genome project (http://congenie.org/). ^*^The intron information is not available.

To identify H3 variants in planta, we performed BLASTP and TBLASTN searches of the complete genomes of eudicots (*Arabidopsis thaliana*,* Capsella rubella*,* Populus trichocarpa*,* Glycine max*, and* Solanum lycopersicum*), monocots (*Zea mays *and* Oryza sativa*), gymnosperm (*Picea abies*), lycophyte (*Selaginella moellendorffii*), bryophyte moss (*Physcomitrella patens*), and chlorophytes (*Volvox carteri*,* Chlamydomonas reinhardtii*,* Ostreococcus lucimarinus*, and* Micromonas pusilla*) (Figure 1). Each matching sequence was then used to search the respective genome databases until no new sequences were found.

After removing incomplete or redundant sequences (nucleic acid sequence redundant) and predicted alternative splice variants, we identified 230 H3 variants (Supplementary Table). The numbers are variable between species (Figure 1). Interestingly, our analysis identified 33 HTR genes in* Chlamydomonas reinhardtii*, most of them were not previously annotated as HTR genes [6]; the variable number of HTR suggested multiple gene loss and gain events during planta evolution.

We first used phylogenetic analysis to define the different H3 groups present in the plant genomes; the phylogenetic tree contains all the H3 variants sequences from (Supplementary Table); the result showed that most of the branches have very low statistics supports (data not shown). However, the clade that contains* Arabidopsis* CenH3 AT1G01370 has good statistics supports, enabling us to identify all the CenH3 variants (Figure 3). All the species investigated have one or two CenH3 gene(s) except the gymnosperm* Picea abies* [29]. One possible explanation is that the recently sequenced Norway spruce genome still has some gaps. In order to discriminate H3.3 from H3.1 in planta, we used previously published criteria [6, 9]: the presence of introns in the H3.3 genes and absence of introns in the H3.1 genes and the four amino acid substitutions commonly found at positions 31, 41, 87, and 90 (T_31_Y_41_H_87_L_90_ for H3.3; A_31_F_41_S_87_A_90_ for H3.1).

**Figure 2:**
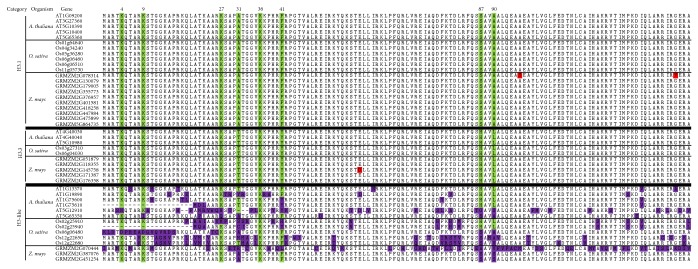
Alignment of* Arabidopsis*, rice, and maize HTR proteins. The signature positions 31, 41, 87, and 90 to distinguish H3.1 and H3.3 are marked. All H3.1 are absence of introns and signature positions are A_31_F_41_S_87_A_90_. All H3.3 contain introns and signature positions are T_31_Y_41_H_87_L_90_. H3-like genes all deviate from H3.1 and H3.3 at positions 31, 41, 87, and 90 and are the presence or absence of introns. The K4, K9, K27, and K36 commonly involved in histone methylations are highly conserved in H3.1 and H3.3. Amino acid substitutions are colored in red (H3.1 and H3.3) and purple (H3-like).

**Figure 3:**
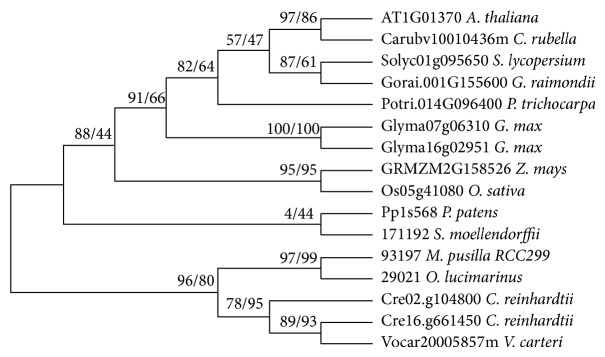
Phylogeny of plant centromeric H3 variants CenH3. For each node, statistical support values are marked (numbers from left to right: neighbor joining inferred under JTT + G models and maximum-likelihood bootstraps inferred using LG + G model).

In our analysis, all members of the H3.3 (Figure 2, Supplementary Table) contain introns and corresponding proteins carry the T_31_Y_41_H_87_L_90_ signature. Members of the H3.1 class are intronless genes and corresponding proteins carry the A_31_F_41_S_87_A_90_ signature (Figure 2, Supplementary Table). For the nonflowering plants, the intronless genes with corresponding proteins carry the A_31_Y_41_(S/Q)_87_L_90_ and we defined them as H3.1A [6]. All the H3-like genes have heterogeneous features [6]: absence or degeneration of N-terminal part of HTR proteins, deviation from H3.1 and H3.3 consensuses at positions 31, 41, 87, and 90, and presence or absence of introns. Our analysis suggested that H3.1, H3-like, and CenH3 already present before the split between chlorophyte and charophyte algae, which is consistent with previous finding [6]. We did not find H3.3 in all the four green algae species, which suggested that H3.3 sequences are more divergent; the signature might be lost in chlorophyta but retained in land plant evolution.

### 3.2. Conserved Characteristics in the H3 Variants

We are particularly interested in grass H3 variants and we performed alignments using HTR protein sequences from* Arabidopsis*, maize, and rice (Figure 2). Analysis revealed nearly identical sequence within H3.1 group and H3.3 group. H3.1 and H3.3 are highly similar except the four signature sites. In* Arabidopsis*, the histone H3 lysine-4 trimethylation (H3K4me3) and H3 lysine-36 di- and trimethylation (H3K36me2/me3) are linked with active gene expression; H3 lysine-9 methylation and H3 lysine-27 trimethylation (H3K27me3) are associated with gene repression [30, 31]. The K4, K9, K27, and K36 are highly conserved in H3.1 and H3.3. K36 is conserved in all the H3 types (Figure 2), indicating that the major lysine posttranslational modifications are conserved. In addition, we observed many substitutions of K4, K9, and K27 in H3-like genes. One interesting substitution is the K27M found in rice Os02g25910. In animal overexpression of histone H3.3K27M results in loss of H3K27 methylation and derepression of polycomb target genes; lysine-to-methionine mutants could inhibit methylation pathways that also function as biochemical reagents for capturing site-specific histone-modifying enzymes [32, 33]. Whether the K27M substitution in rice is involved in inhibiting K27 methylation will be a subject for further investigation.

We found that the HTR genes in* Capsella rubella *are significantly different from all the other species' HTRs. We performed an alignment compared with* Arabidopsis thaliana* (Supplementary Figure  1). The* Capsella rubella* HTRs were highly conserved within the species. However, even though* Capsella rubella* is the close relative to* Arabidopsis*, we did not find the H3.1 or H3.1 signature. Furthermore, we observed multiple deletions, the deletions were found in all the H3-like genes except CenH3, and the corresponding sequences were highly conserved in* Arabidopsis*. This suggested that CenH3 evolved independently to other H3 variants; the deletion event happened before the duplication of H3-like variants in* Capsella rubella*.

### 3.3. Molecular Evolution of Plant HTR Genes

Comparing rates of *dN* and *dS* is a common way to examine selection pressures on coding regions. Commonly, a *dN*/*dS* value of 1 is used to indicate neutral selection and values lesser or greater than 1 to, respectively, indicate purifying and positive selection [34]. To analyze the selective pressures acting during the expansion of plant HTR genes, we investigated the influences of selective constraints on the three group H3 variants coding region. By globally fitting an evolutionary model, we first calculated the *dN*/*dS* ratios for each group. The *dN*/*dS* values were substantially <1 in all groups, providing a crude indication that the strong purifying selection has been maintained across plants, implying the H3 variants functional conservation. At the individual codon level, most of the residues were under significant negative selection (*P* < 0.05) (Supplementary Figure  2). We also observed several sites under relaxed constrain (Supplementary Figure  2), which may contribute to the functional divergent [9].

### 3.4. Expression Analysis of HTR Genes at Different Developmental Stages

To understand the temporal and spatial expression patterns of grass HTR genes, we compared their expression patterns during maize and rice development.

**Figure 4:**
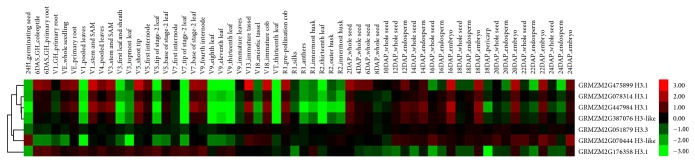
Expression profiles of HTR genes in maize across different developmental stages and organs. The genes IDs are on the right. The tissues used for expression analysis are indicated at the top of each column. The color bar represents log2 expression values.

Microarray data of 60 different tissues and developmental conditions of maize were used [24]. Several genes were not detected in this dataset, suggesting that they might be pseudogenes. The expressed genes were detected in all samples examined (Figure 4). The two H3.3 genes showed different expression patterns: H3.3 GRMZM2G051879 was constitutively expressed in all organs and developmental stages; in contrast, H3.3 gene GRMZM2G176358 was highly expressed in all stage roots and leaves, but low expressed in seeds, embryo, and endosperm. All the H3.1 genes (GRMZM2G475899, GRMZM2G078314, GRMZM2G447984) showed higher expression in roots, stem, seed, and embryo, but lower expression in all stages of leaves and endosperm, which suggested they play important roles in root, meristem, and embryo development. The H3-like gene GRMZM2G387076 has similar expression to H3.1. GRMZM2G387076 is intronless also suggest that this gene is evolutionary more close to H3.1. The other H3-like gene GRMZM2G070444 contains intron; its expression has heterogeneous feature: low expressed in roots and leaves and highly expressed in seed, embryo, and endosperm.

**Figure 5:**
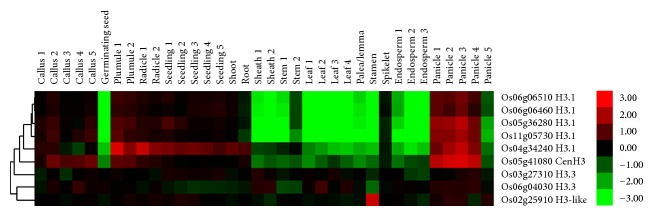
Expression profiles of HTR genes in rice across different developmental stages and organs. The genes IDs are on the right. The tissues used for expression analysis are indicated at the top of each column. The color bar represents log2 expression values.

We next analysed the expression profiles of rice HTR genes (Figure 5). The genome arrays from 39 tissues collected throughout the life cycle of the rice were used [25]. The H3.3 genes were highly expressed in majority samples; the intron-containing H3-like gene LOC_Os02g25910 has similar expression patterns with H3.3. All the H3.1 genes have the same expression features: they expressed at higher level in callus, seedlings, shoot, and root and lower level in leaves, endosperms, and so forth, which is highly similar with maize, indicating functional conservation of HTRs in plants.

### 3.5. Functional Conservation and Diversity of HTR Genes

The comparative phylogenetic analysis of plant HTR proteins enables us only to identify the CenH3 variants. Therefore, we performed alignment to classify H3.1, H3.3, and H3-like variants using the established signatures [6]. In* Arabidopsis*, the H3.1 genes showed high level expression in tissues containing rapidly dividing cells; the H3.3 genes exhibited high level of expression in most of the tissues examined.* Arabidopsis* H3.1 and H3.3 were proposed as replication dependent and replication independent, respectively [9]. Our expression profiles showed the H3.1 and H3.3 expression was similar among maize, rice, and* Arabidopsis *[9], indicating the function conservation in angiosperm. The purifying selection also supports the functional constraint during plant evolution. Collectively, these findings indicate that in flowering plant H3.1 variants are replication dependent and the H3.3 variants are replication independent.

Our analysis identified a big number of H3-like genes. The numbers of H3-like genes are different between species. The H3-like genes' expression patterns are similar with H3.1 or H3.3 or have heterogeneous feature [9] (Figures 4 and 5). In* Arabidopsis*, the H3-like gene At1g19890 is male gamete specific; disruption of this gene might be compensated by other H3 genes [9]. Therefore, the H3-like genes may be full/partial redundancy to other HTRs or have a dosage dependent manner when forming nucleosome. Recent paper showed that the histone concentrations affect gene expression through nucleosome repeat length (NRL) [35]. Considering the number of H3-like genes in each species may affect the histone concentration; it is tempting to hypothesize that H3-like variants contribute to control gene expression by regulating NRL. Null mutants of H3-like genes are required to test the hypothesis.

## 4. Conclusion

Histone H3 variants in animals and* Arabidopsis* are known to be crucial for a multitude of physiological and intracellular processes. Here, we report an identification, evolution, and expression of the H3 variants in plants; the comparative genomic analyses of H3 variants establish a framework for understanding the evolutionary mechanisms involved in the origin and expansion of plant HTR genes, and it provides a basis for investigating cellular functions of HTR genes.

## Supplementary Material

Supplementary Figure 1: Alignment of Arabidopsis thaliana and *Capsella rubella* HTR proteins. *Capsella rubella* HTRs have several conserved deletions. The H3.1 or H3.1 signature are absent in *Capsella rubella* HTR proteins.
Supplementary Figure 2: Analysis of site-specific levels of selection pressure in H3.3 (A) and H3.1(B) proteins. Most of the residues were under significant negative selection (*P*< 12 0.05) and several sites under relaxed constrain(A). The x-axis numbers correspond to the numbering of the amino acids in the Arabidopsis thaliana H3.3 (A) and H3.1(B). The y-axis values are the ratio of dN/dS.
Supplementary Table: Accession numbers of HTR genes used in this study.
