# Supplementary figures and images for: Genome-Wide Identification, Evolutionary, and Expression Analyses of Histone H3 Variants in Plants
Source: Biomed Res Int. 2015 Feb 26;2015:341598. doi: 10.1155/2015/341598 (PMC4357034; doi:10.1155/2015/341598)

A

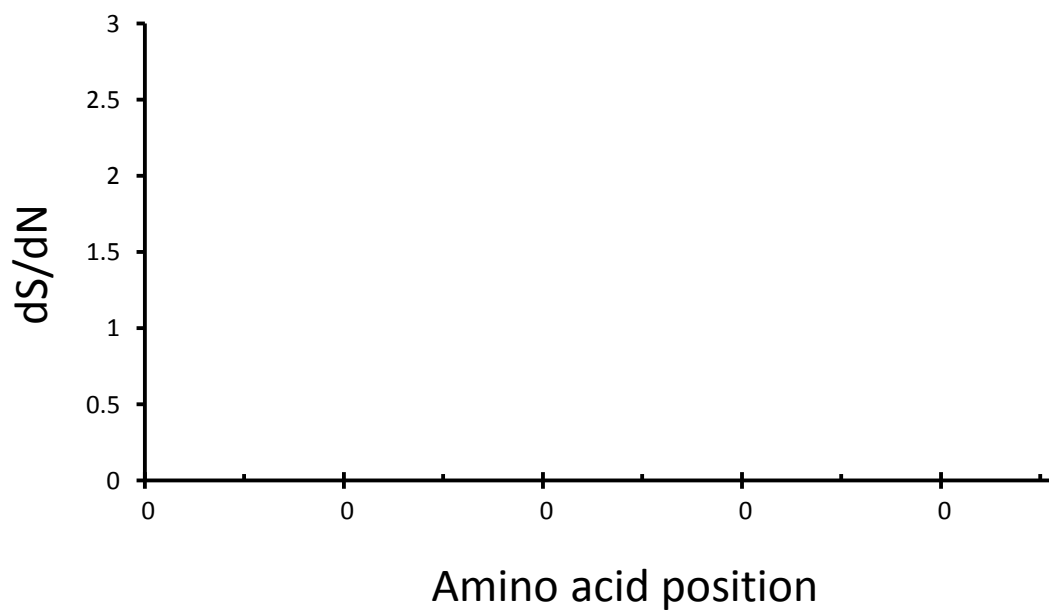

B

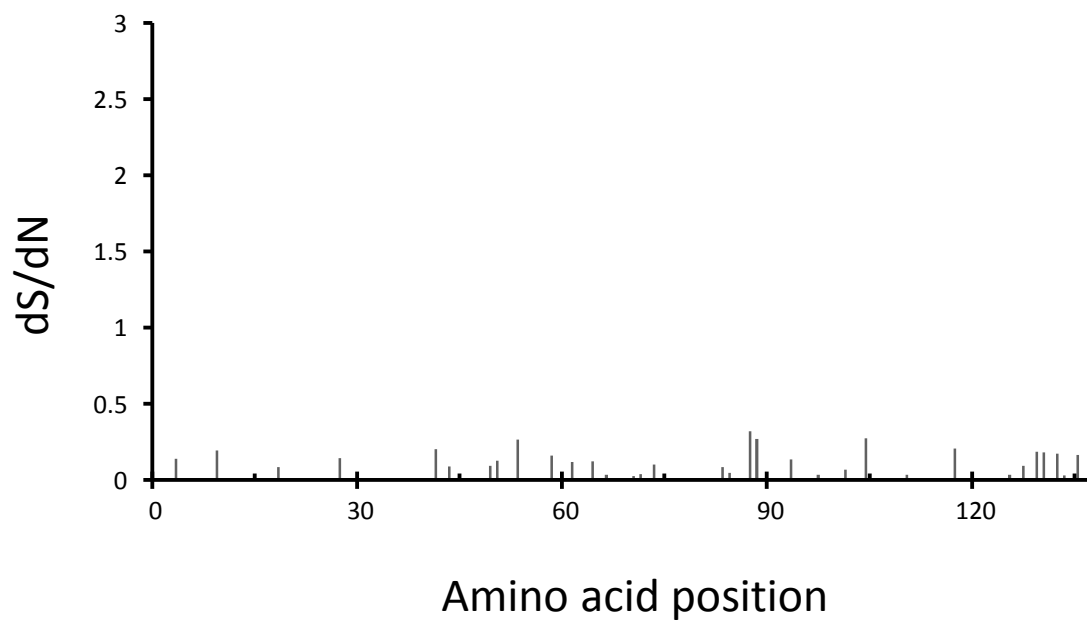

Supplement: Supplementary file 2 [file 341598.f2.pdf]
